# Foreign Body Immune Response to Zwitterionic and Hyaluronic Acid Granular Hydrogels Made with Mechanical Fragmentation

**DOI:** 10.1002/adhm.202402890

**Published:** 2024-11-05

**Authors:** Maryam Asadikorayem, Patrick Weber, František Surman, Anna Puiggalí‐Jou, Marcy Zenobi‐Wong

**Affiliations:** ^1^ Tissue Engineering + Biofabrication Laboratory, Department of Health Sciences and Technology ETH Zürich Otto‐Stern‐Weg 7 Zürich 8093 Switzerland

**Keywords:** fragmentation, granular, hyaluronic acid, hydrogels, immune response, microgels, zwitterionic

## Abstract

Granular hydrogels have recently attracted the attention for diverse tissue engineering applications due to their versatility and modularity. Despite previous studies showing enhanced viability and metabolism of cells encapsulated in these hydrogels, the in vitro immune response and long‐term fibrotic response of these scaffolds have not been well characterized. Here, bulk and granular hydrogels are studied based on synthetic zwitterionic (ZI) and natural polysaccharide hyaluronic acid (HA) made with mechanical fragmentation. In vitro, immunomodulatory studies show an increased stimulatory effect of HA granular hydrogels compared to bulk, while both bulk and granular ZI hydrogels do not induce an inflammatory response. Subcutaneous implantation in mice shows that both ZI and HA granular hydrogels resulted in less collagen capsule deposition around implants compared to bulk HA hydrogels 10 weeks after implantation. Moreover, the HA granular hydrogels are infiltrated by host cells, including macrophages and mature blood vessels, in a porosity‐dependent manner. However, a large number of cells, including multinucleated giant cells as well as blood vessels, surround bulk and granular ZI hydrogels and are not able to infiltrate. Overall, this study provides new insights on the long‐term stability and fibrotic response of granular hydrogels, paving the way for future studies and applications.

## Graphical abstract

This study explores the in vitro and long‐term in vivo immune responses to microporous granular hydrogels based on zwitterionic carboxybetaine acrylamide and hyaluronic acid. The findings reveal biomaterial‐ and porosity‐dependent effects on in vitro immunostimulatory and in vivo fibrotic responses. Zwitterionic granular hydrogels are surrounded by an inflammatory cell layer, while hyaluronic acid granular hydrogels support cell and tissue infiltration.

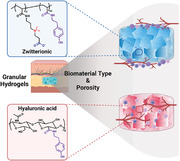

## 1 Introduction

Host immune response to biomaterials designed for clinical applications is a key parameter determining their successful translation.^[^ ^1^ ^]^ An uncontrolled immune response can result in fibrous encapsulation of the implants through a process called foreign body response (FBR), resulting in implant isolation and failure.^[^ ^2^ ^]^ However, a controlled immune response is a pre‐requisite for wound healing and tissue repair to modulate biomaterial‐host tissue integration.^[^ ^3^ ^]^ Understanding and modulating the immune response to biomaterials is therefore an essential and powerful tool to optimize them for different applications. Numerous design parameters have been studied and varied to modulate in vivo biocompatibility and immune response of biomaterials.^[^ ^4^ ^]^ Surface topography,^[^ ^5^ ^]^ surface charge,^[^ ^6^ ^]^ implant size and shape,^[^ ^7^ ^]^ and implant stiffness,^[^ ^8^ ^]^ have been among the most researched parameters.

Tuning material porosity and pore size has additionally shown promise in modulating immune response toward enhanced tissue integration in vivo.^[^ ^9^ ^]^ Hydrogels with an interconnected porosity at the scale of cells facilitate cell mobility, diffusion of biological factors, and ingrowth of vasculature and nascent tissue.^[^ ^10^ ^]^ Granular hydrogels, made of packed or annealed microgels, are a class of versatile porous scaffolds with emerging applications in tissue engineering.^[^ ^11^ ^]^ In recent years, these scaffolds have been explored as in vitro cell culture platforms, for the delivery of biologics as well as for in vivo tissue regeneration applications.^[^ ^12^ ^]^ The bottom‐up design of granular hydrogels offers many appealing characteristics, such as injectability, printability, and heterogeneity.^[^ ^13^ ^]^ As granular hydrogels become more widely utilized, understanding how these scaffolds modulate immune responses both in vitro and in vivo can empower more tailored designs and inspire future applications. Segura and co‐workers have shown that granular hydrogels made of spherical microgels improve tissue closure and vascularization in wound healing models compared to bulk scaffolds.^[^ ^14^ ^]^ Also, recently they showed that the pore size of these granular hydrogels can direct macrophage response and wound healing outcomes in vivo.^[^ ^15^ ^]^ The effect of microgel shape on in vivo response to granular hydrogels has also been studied, showing increased and more homogenous cell infiltration and vessel invasion into granular hydrogels made with rod‐shaped particles compared to spherical ones.^[^ ^16^ ^]^

The constituent microgels in granular hydrogels can be produced by forming droplets prior to crosslinking, such as in microfluidics setups,^[^ ^17^ ^]^ or by fragmenting crosslinked bulk hydrogels into smaller pieces.^[^ ^18^ ^]^ The microgel fabrication method has a strong effect on microgel shape and size as well as subsequent granular hydrogel properties.^[^ ^19^ ^]^ Mechanical fragmentation of hydrogels is a simple, tunable, and scalable method that does not require the use of any toxic materials or complicated setups and is applicable to a wide range of hydrogels.^[^ ^18^ ^]^ This method results in irregularly shaped microgels with a wide microgel size distribution.^[^ ^20^ ^]^ Moreover, the resulting granular hydrogel supports long‐term cell culture, as it has higher mechanical stability compared to other microgel fabrication techniques.^[^ ^20^ ^]^ In recent years, granular hydrogels made with mechanical fragmentation have been used for the engineering of diverse tissues such as for articular^[^ ^21^ ^]^ and auricular^[^ ^22^ ^]^ cartilage, meniscus,^[^ ^23^ ^]^ intervertebral disc,^[^ ^24^ ^]^ spinal cord,^[^ ^25^ ^]^ and muscle^[^ ^26^ ^]^ regeneration as well as for delivery of biologics.^[^ ^27^ ^]^ However, the in vitro immune response as well as long‐term in vivo fibrotic response to these versatile granular hydrogels has not been well characterized.

Besides hydrogel architecture, chemical composition can also play a big role in tuning the immune response. Zwitterionic hydrogels, having equal amounts of cationic and anionic moieties, have been shown to be anti‐fouling and cell‐repellant and to effectively inhibit fibrotic response and collagen capsule formation in vivo.^[^ ^28^ ^]^ However, all of the previous studies used bulk hydrogels, which are nanoporous and have limited applicability in tissue regeneration. We have recently developed zwitterionic granular hydrogels that are microporous, injectable, and printable and allow for direct encapsulation of cells, supporting their viability and extracellular matrix deposition.^[^ ^29^ ^]^ Here we aim to study how the long‐term in vivo fibrotic response to this more versatile zwitterionic granular hydrogel differs compared to bulk. On the other hand, hyaluronic acid is one of the most abundantly used natural biomaterials in tissue engineering, that encourages cell interactions and scaffold remodeling,^[^ ^30^ ^]^ and has been shown to have immunostimulatory effects.^[^ ^31^ ^]^ While there are many studies on granular hydrogels made with hyaluronic acid, the fibrotic in vivo immune response to these porous hydrogels remain understudied.

**Figure 1:**
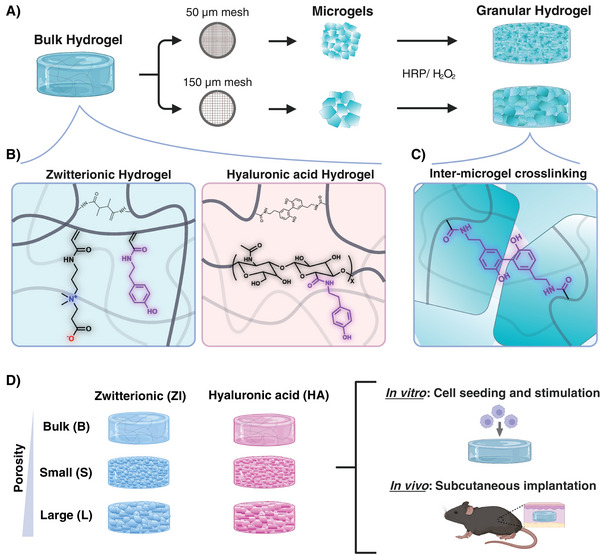
Illustration of the biomaterial synthesis and study plan. A) Granular hydrogels are made with mechanical fragmentation of bulk hydrogel using 50 or 150 µm grids. The resulting microgels with two different sizes are then secondarily crosslinked using horseradish peroxidase (HRP) and hydrogen peroxide (H_2_O_2_) to produce microporous granular hydrogels. B) The composition of the two bulk hydrogels; Zwitterionic (ZI) hydrogel is formed through photopolymerization of carboxybetaine acrylamide using gelatin methacrylate, and the hyaluronic acid (HA) hydrogel is crosslinked enzymatically via HRP. Both hydrogels contain functional tyramine moieties, shown in purple, which enable secondary crosslinking of the microgels. C) Inter‐microgel covalent bond formation via HRP/H_2_O_2_ to create stable microporous scaffolds. D) The six hydrogel groups used in the study, with different biomaterials and porosities and schematics of the in vitro and in vivo experiments. Illustration created with BioRender.com.

Overall, the aim of this study is to understand how the in vitro and in vivo immune responses to granular hydrogels made with mechanical fragmentation differ from the responses to bulk hydrogels, as well as what is the effect of porosity and biomaterial (**Figure** **1**A). To this end, granular hydrogels with two different biomaterials, zwitterionic (ZI) carboxybetaine acrylamide and hyaluronic acid (HA), each with two different porosities was made. (Figure 1B). The choice of HA in this study is partly motivated by its widespread use in the literature on granular hydrogels, particularly those with in vivo studies,^[^ ^16^, ^22^, ^32^ ^]^ though with varying formulations, microgel shapes, and animal models. This body of research provides a useful foundation for our analysis. Additionally, comparing zwitterionic materials with HA, each having distinct roles in tissue engineering, provides further insights into their respective potential for future applications. To achieve two different porosities of granular hydrogels, two grid sizes of 50 and 150 µm were used for hydrogel fragmentation. The microgels produced from fragmentation processes are secondarily crosslinked to form a stable microporous granular hydrogel through inter‐microgel enzymatic crosslinking using horseradish peroxidase (HRP) and hydrogen peroxide (H_2_O_2_), forming covalent bonds between tyramine moieties in the microgels (Figure 1C). It has been shown that HRP‐mediated crosslinking is biocompatible and stable in vivo.^[^ ^33^ ^]^ Moreover, we have previously shown that this crosslinking method can quickly stabilize microgels and form scaffolds that are stable for a long period,^[^ ^29^ ^]^ eliminating the need for an interstitial matrix which can compromise microporosity.^[^ ^34^ ^]^ The hydrogels with different biomaterials and porosities were then tested both in vitro and in vivo (Figure 1D). In vitro experiments were done by seeding immune cells on the scaffolds, with and without additional stimulation, and investigating the activation of inflammatory pathways. In vivo experiment was done by subcutaneous implantation of hydrogels in immunocompetent mice for 10 weeks to analyze long‐term tissue integration, fibrosis, and stability of the hydrogels. Overall, this study provides new insights into how granular hydrogels with different biomaterials and porosities can modulate in vitro and in vivo immune responses.

## 2 Results

### 2.1 Fabrication of Zwitterionic and Hyaluronic Acid Granular Hydrogels with Matched Porosities and Mechanical Properties

Granular hydrogels were fabricated with mechanical fragmentation technique using a custom‐made extruder (Figure S1, Supporting Information), in which a bulk hydrogel is successively fragmented into microgels by pressing it through micron‐sized metal grids. This method results in irregularly shaped microgels with average microgel size controlled by the grid size used for fragmentation. Granular hydrogels made by mechanically fragmented microgels are injectable and printable by extrusion 3D printing,^[^ ^23^ ^]^ and can withstand high levels of strain.^[^ ^29^ ^]^ Furthermore, they can promote cell migration and endogenous tissue repair^[^ ^21^ ^]^ and support long‐term in vitro cell culture.^[^ ^22^ ^]^ In this study, granular hydrogels were fabricated with either zwitterionic (ZI) or hyaluronic acid (HA). First, bulk ZI and HA hydrogels were produced for fragmentation and as controls throughout the study. ZI hydrogels were produced by photopolymerization of a zwitterionic carboxybetaine acrylamide (CBAA) monomer together with a tyramine acrylamide (TyrAA) comonomer using gelatin methacryloyl (GelMA) as a biocompatible crosslinker. HA hydrogels were fabricated by enzymatic crosslinking of hyaluronic acid tyramine (HATyr) using HRP and H_2_O_2_. The remaining unreacted tyramine moieties in the HA bulk hydrogels were sufficient for inter‐microgel enzymatic crosslinking of the resulting microgels.^[^ ^22^ ^]^ It is important to note that while we employ different chemistries for the initial bulk hydrogel fabrication, the secondary crosslinking mechanism (enzymatic) for annealing the microgels is identical. We believe this secondary mechanism has the most integral role in defining the properties of the granular hydrogels and their interactions with cells. While the initial crosslinking might influence the (nano)porosity of the bulk hydrogels, given that cells are not encapsulated within this matrix, we believe that with an identical secondary annealing mechanism, porosity and material chemistry (ZI vs HA) will be the main determinants in cell‐biomaterial interactions. However, the initial crosslinking mechanism and network will have an impact on matrix remodeling and degradation.

**Figure 2:**
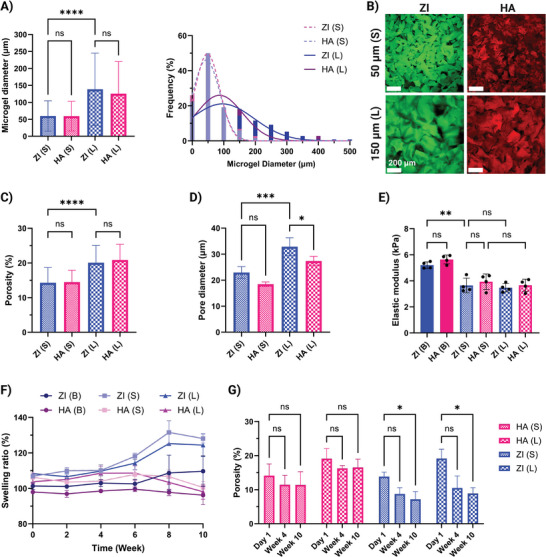
Microgels and granular hydrogels characterization. A) Average diameter of microgels after fragmentation with 50 (S) and 150 (L) µm grid and microgel size distribution (*n* = 250 microgels). B) Confocal images of labeled granular hydrogels after secondary enzymatic crosslinking (Scale bar 200 µm). C) Average porosity of granular hydrogels (*n* = 3 granular hydrogels, 10 images per hydrogel). D) Average pore diameter of granular hydrogels (*n* = 3 granular hydrogels, 10 images per hydrogel). E) Elastic modulus of hydrogels calculated from the linear region of stress–strain curve (*n* = 4). F) Swelling ratio of hydrogels during 10 weeks of incubation in PBS (*n* = 3). G) Average porosity of granular hydrogels over 10 weeks of incubation in PBS (*n* = 3 granular hydrogels, 10 images per hydrogel). Data are represented as mean ± standard deviation. Statistical significance was determined using a one‐way (A, D, and E) or two‐way (G) ANOVA with a Tukey's multiple comparisons test (non‐significant (ns) *p* > .05, ^∗^ *p* < .05, ^∗∗^ *p* < .01, and ^∗∗∗∗^ *p* < .0001).

The bulk hydrogels were then mechanically pressed through 50 or 150 µm aperture metal grids.^[^ ^18^ ^]^ As shown in **Figure** **2**A, the material type had no influence on microgel size, and only the grid size controlled average microgel size as well as size distribution. Microgels made with 50 µm grid had an average size of ≈59 ± 44 µm and those made with 150 µm grid had an average size of ≈130 ± 100 µm. Additionally, a comparison of the average size and size distribution of the microgels produced in this study to those with similar zwitterionic materials and sizing setup from our previous work demonstrates low batch‐to‐batch variation in the method.^[^ ^29^ ^]^ The microgels from each material type were reswollen at a concentration matching the equilibrium water content of the corresponding bulk hydrogels (Figure S2, Supporting Information), to inhibit excessive swelling or shrinking of the resulting granular hydrogel. The microgel slurries were then cast in cylindrical PDMS molds and crosslinked with HRP and H_2_O_2_, resulting in granular hydrogel discs (Figure S1, Supporting Information).

The microstructure of the resulting granular hydrogels for the two material types and two grid sizes are shown in Figure 2B. As shown in previous studies,^[^ ^29^ ^]^ a larger grid size resulted in a larger overall porosity of the granular hydrogels. It should be noted that the granular hydrogels made with the two material types had similar overall porosities depending only on the grid size. The ZI and HA granular hydrogels made with 50 µm grid had 13.99 ± 4.60% and 14.51 ± 3.99% porosities, and the ones made with 150 µm grid had 19.81 ± 5.08% and 21.18 ± 4.74% porosities, respectively (Figure 2C). The average diameter of the pores was also approximated from confocal images showing pores with ≈20 and 30 µm diameter for granular hydrogels made with small and large microgels respectively, with slightly smaller pores measured in HA scaffolds (Figure 2D). Also, pore interconnectivity within granular hydrogels was evaluated following the reported protocol to calculate tortuosity, by simulating the displacement of an array of small beads and tracking their trajectory through the scaffold.^[^ ^16^ ^]^ The analysis showed that the majority of beads could cross through the scaffolds, reconstructed from confocal images, indicating interconnectivity. Moreover, there was a shorter path for smaller microgels with no difference between the two materials (Figure S3, Supporting Information). Scanning electron microscopy (SEM) also showed how the surface and inner structure of bulk and granular hydrogels differ, showing the micropores between microgels in granular hydrogels (Figure S4, Supporting Information). However, SEM imaging introduces some artifacts to hydrogels, due to the drying process, making quantitative measurements more difficult.^[^ ^35^ ^]^ We also tested the mechanical properties of both bulk and granular hydrogels by compression testing. The concentrations of materials for bulk hydrogel fabrication were initially tuned, as described in the methods section so that the bulk hydrogels had similar elastic modules (5.20 ± 0.25 and 5.64 ± 0.33 kPa for ZI and HA hydrogels respectively) (Figure 2E). Moreover, hydrogels with the same porosity also had a similar elastic modulus (3.64 ± 0.56 and 3.93 ± 0.59 kPa for ZI and HA hydrogels made with the 50 µm grid and 3.47 ± 0.32 and 3.66 ± 0.46 for the ones made with the 150 µm grid, respectively) (Figure 2E). The elastic modulus for hydrogels with larger porosity was slightly lower than the ones with smaller porosity, but the difference was not significant.

As the in vivo experiment is planned for a long‐term response after 10 weeks of implantation, we also performed long‐term in vitro stability analysis looking at swelling ratio and porosity changes over time. The bulk and granular hydrogel discs were kept in PBS at 37 °C for 10 weeks and samples were weighed every other week. As shown in Figure 2F, all samples were stable and there was no degradation or disintegration. However, ZI granular hydrogels had higher swelling ratios overall, especially at later timepoints after 6 weeks. We also took confocal images at 4 and 10 weeks and calculated the overall porosity of the granular scaffolds after a long incubation time. In all cases, granular hydrogels preserved interconnected porous structures at both 4 and 10 week timepoints (Figure S5, Supporting Information). However, the overall porosity decreased over time, due to swelling of microgels (Figure 2G). The decrease in porosity was more significant for ZI granular hydrogels reaching ≈7 and 9% for ZI small and large microgels compared to ≈11 and 15% for HA small and large microgels respectively after 10 weeks. This represents an average reduction in porosity of ≈50% for ZI granular hydrogels, in contrast to a 20% reduction for HA granular hydrogels. The greater decrease in porosity for ZI granular hydrogels aligns with their higher swelling ratio, which fills the inter‐microgel spaces. Moreover, we also performed in vitro enzymatic degradation in collagenase and hyaluronidase for ZI and HA granular hydrogels respectively. We observed that the degradative property of HA was preserved in our synthesized HA‐Tyr resulting in fast degradation of both bulk and granular HA hydrogels (Figure S6A, Supporting Information). However, while collagenase incubation resulted in fast degradation of GelMA‐crosslinked ZI bulk hydrogels, the ZI granular hydrogels only showed an increased swelling ratio after 6 days compared to PBS incubation and not a fast degradation profile (Figure S6B, Supporting Information). This differential degradation behavior of ZI and HA could also contribute to the differences in swelling and porosity changes over time. Further investigation into the degradation kinetics, including long‐term changes in material mass along porosity variations, would provide a clearer understanding of the underlying mechanisms.

### 2.2 Mechanical Fragmentation Increases Pro‐Inflammatory Effect of Hyaluronic Acid Hydrogels In Vitro but has no Effect on Zwitterionic Hydrogels

**Figure 3:**
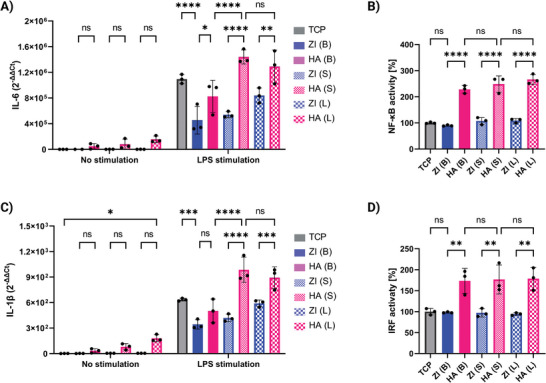
In vitro immune response. A) IL‐6 and C) IL‐1β gene expression for THP‐1 cells seeded on hydrogels for 24 hrs. SEAP reporter readout for B) nuclear factor kappa B (NF‐κB) and D) interferon regulatory factor (IRF) activation for THP‐1 cells seeded on hydrogels for 24 hrs. Data are represented as mean ± standard deviation. Statistical significance was determined using a one‐way (B and D) or two‐way (A and C) ANOVA with a Tukey's multiple comparisons test (non‐significant (ns) *p* > .05, ^∗^ *p* < .05, ^∗∗^ *p* < .01, ^∗∗∗^ *p* < .001, and ^∗∗∗∗^ *p* < .0001). (*n* = 3 replicates).

The in vitro immune response to biomaterials using macrophages and other immune cells has been used to predict the in vivo response and the overall biocompatibility of biomaterials used for biomedical applications.^[^ ^36^ ^]^ To overcome issues of limited lifespan and variability of monocyte‐derived human macrophages, the THP‐1 acute monocytic leukemia cell line is frequently used for in vitro immunomodulatory studies.^[^ ^37^ ^]^ THP‐1 cells exhibit many similarities to primary monocytes with respect to cell morphology, expression of membrane receptors and antigens as well as secretory products.^[^ ^38^ ^]^ To simulate the encounter of immune cells with biomaterials after implantation, we seeded THP‐1 cells on our six hydrogel types either with no further stimulation or in the presence of 100 ng mL^−1^ lipopolysaccharide (LPS) as a stimulatory factor. We looked at expressions of the inflammatory genes interleukin 6 (IL‐6) and interleukin 1 beta (IL‐1β) 24 hrs after seeding. As shown in **Figure** **3**A,C, ZI bulk and granular hydrogels had no stimulatory effect on THP‐1 cells, while HA hydrogels induced increased IL‐6 and IL‐1β gene expression, even with no LPS stimulation. In the presence of LPS stimulation, both bulk and granular ZI hydrogels were again non‐immunogenic; additionally, the ZI bulk hydrogel resulted in reduced inflammatory response compared to tissue culture plastic (TCP). On the other hand, while HA bulk hydrogel was also non‐stimulatory, granular HA hydrogels resulted in an increased inflammatory response compared to HA bulk and TCP. Additionally, there is a noticeable trend of increased stimulatory effect with HA granular hydrogels composed of smaller microgels compared to larger ones, which could be attributed to the increased surface area of smaller particles.

The nuclear factor kappa B (NF‐κB) and the interferon regulatory factor (IRF) transcription factor families are major players in inflammation and act as effectors of the innate immune response.^[^ ^39^ ^]^ Moreover, IL‐6 and IL‐1β are both known to be downstream effectors of NF‐κB activation.^[^ ^40^ ^]^ The THP‐1 cells used in this study have two inducible cell‐based reporter genes, which allow for the simultaneous study of NF‐κB and IRF pathways, by monitoring the activity of secreted embryonic alkaline phosphatase (SEAP) and Lucia luciferase in the media. We observed significant activation of both pathways in the presence of HA bulk and granular hydrogels (Figure 3B,D), while the ZI hydrogels showed no stimulatory effect compared to TCP. We also differentiated THP‐1 cells to adherent macrophages using phorbol 12‐myristate 13‐acetate (PMA).^[^ ^41^ ^]^ More macrophages were able to adhere to HA hydrogels compared to the ZI hydrogels after 24 hrs of seeding (Figure S7, Supporting Information).^[^ ^42^ ^]^ Also, the number of attached macrophages was increased in granular hydrogels compared to bulk. It has been previously shown that there is a direct correlation between macrophage attachment to an implant and the subsequent FBR in vivo.^[^ ^43^ ^]^ Moreover, the trend for IL‐6 and IL‐1β gene expression and for NF‐κB and IRF activation for these differentiated cells was similar to undifferentiated THP‐1 cells, showing more inflammatory response for HA compared to ZI. (Figure S8, Supporting Information). The response from differentiated adherent cells was generally stronger, likely due to their primed state and their stronger interactions with the materials, as a result of their adherent nature.

Our in vitro results indicate that HA hydrogels overall exhibit a higher stimulatory effect on THP‐1 cells compared to ZI hydrogels, with mechanical fragmentation further increasing this effect. On the other hand, we observe that the mechanical fragmentation of ZI hydrogels does not affect their in vitro stimulatory effect. Moreover, in this study, we did not observe significant differences between different porosities of granular hydrogels. It might be due to the fact that the designed experiment is rather a 2D experiment, where cells are seeded on top of the constructs, than a 3D encapsulation, as the aim was to simulate immune cell encounter with hydrogel surface.

### 2.3 Host Cells Infiltrate Hyaluronic Acid Granular Hydrogels, while Zwitterionic Granular Hydrogels are Surrounded by a Layer of Multinucleated Giant Cells

Next, we investigated the effect of biomaterial and porosity of our bulk and granular hydrogels on their long‐term in vivo compatibility and FBR. We implanted the six hydrogel types (Figure 1) subcutaneously in immunocompetent C57BL/6 mice. Hydrogel discs of 10 mm in diameter and 3 mm in height were implanted under the skin dorsally, and after 10 weeks, the samples and the surrounding tissue were harvested for histological analysis. During the experiment and upon sample retrieval, no sign of excess inflammation was observed, and samples were still visible under the skin at explantation day (Figure S9, Supporting Information). After explantation, bulk hydrogels as well as ZI granular hydrogels had retained their shape, while HA granular hydrogels, and especially the ones with larger porosity looked a bit deformed in some animals. Unlike the ZI implants and HA bulk hydrogels that preserved their original disc structure, the HA granular hydrogels became misshaped and distorted (Figure S9, Supporting Information). The hydrogels and the surrounding skin were processed for histological and immunohistochemical analyses, including H&E staining for overall tissue morphology and host cell invasion, Masson's trichrome staining for collagen deposition, and immunostainings for CD68 (macrophages) and CD31 (endothelial cells) markers.

**Figure 4:**
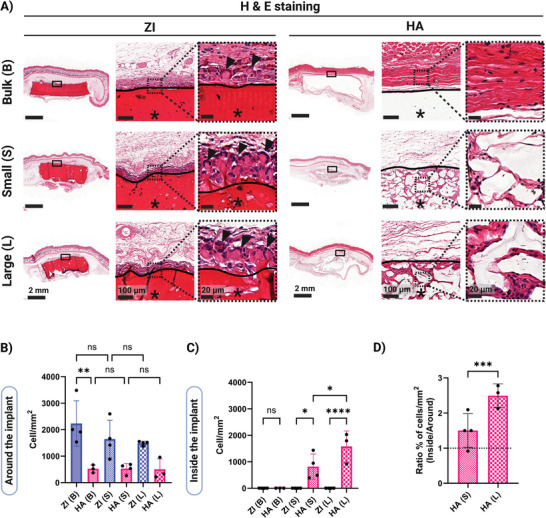
Host cell population around and inside the implants 10 weeks after subcutaneous implantation. A) Representative hematoxylin and eosin (H&E) staining images (scale bars: 2 mm, 100 µm and 20 µm from left to right zoom‐in images; black line indicates hydrogel interface with animal tissue, asterisks (*) indicate the location of the implanted hydrogel and black arrow indicates multinucleated giant cells (MNGCs)). B,C) Quantification of cell nuclei density at an area adjacent to the implants and inside the implants. D) The ratio of cells inside and around the HA granular hydrogels. Data are represented as mean ± standard deviation. Statistical significance was determined using a one‐way ANOVA with a Tukey's multiple comparisons test (non‐significant (ns) *p* > .05, ^∗^ *p* < .05, ^∗∗^ *p* < .01, and ^∗∗∗∗^ *p* < .0001). (*n* = 4 replicates).

As anticipated from macroscopic observations, morphological examination of the implants through H&E staining revealed intact structures for ZI and HA bulk and ZI granular hydrogels, while HA granular hydrogels displayed a more deformed architecture (**Figure** **4**). We observed striking differences in cell populations and tissue ingrowth both around and inside the implants based on biomaterial and porosity (Figure 4A; Figure S10, Supporting Information). As expected, both ZI and HA bulk hydrogels remained intact and acellular, resisting tissue ingrowth. However, while HA granular hydrogels were fully populated with endogenous cells and tissue at the pores between microgels, ZI granular hydrogels, having similar initial porosities to HA granular hydrogels, showed minimal to no cell infiltration. Very minor and inconsistent areas of cell infiltration into ZI granular hydrogels were observed at the implant‐tissue interface which could be due to mechanical breakdown of the scaffold during implantation, rather than consistent cell infiltration. Also, ZI granular hydrogels showed a tighter packing compared to HA granular hydrogels in all the stainings, due to lack of tissue ingrowth. Regarding cells at the implant‐host tissue interface, we observed an inflammatory cell layer including many multinucleated giant cells (MNGCs), surrounding all ZI hydrogels, while there was low cellularity around all the HA hydrogels (Figure 4A). There was no significant difference in cell densities around implants for different microstructures made with the same biomaterial (Figure 4B). However, there were in general ≈4 times more cells at implant‐host tissue interface of ZI scaffolds compared to HA scaffolds (Figure 4B). The HA bulk scaffold was surrounded by dense collagenous tissue which generally has low cellularity.^[^ ^44^ ^]^ Moreover, as cells were able to infiltrate into HA granular scaffolds, this can also partially contribute to a lower cell density around these scaffolds compared to ZI granular scaffolds, where cells did not infiltrate.

An interesting and significant observation in this study was the many MNGCs at the implant‐host tissue interface of all the ZI scaffolds, and especially ZI granular scaffolds as indicated by black arrows in Figure 4A. MNGCs are believed to be the result of macrophage fusion, normally in an attempt to accelerate foreign material phagocytosis and degradation.^[^ ^45^ ^]^ These cells are associated with foreign body response to implants as well as with more angiogenesis and blood vessel formation.^[^ ^46^ ^]^ Moreover, the presence of these cells at different timepoints after implantation has been associated with the degradation kinetics of implants, with slower degradation‐inducing persistence presence of MNGCs at longer time points.^[^ ^47^ ^]^ As our ZI granular scaffolds, which are initially crosslinked by GelMA, are prone to a very slow degradation in the presence of collagenase, this might partially contribute to this observation.^[^ ^29^ ^]^ Also, the more pronounced presence of MNGCs around ZI granular hydrogels compared to bulk could be due to the increased surface area and possible release of micrometer‐sized particulates stimulating macrophages. It has been suggested that the temporal trend of the MNGCs population over time is also important in determining the eventual response and fate of the implant.^[^ ^48^ ^]^ Thus, it is critical to study both earlier and later timepoints to better understand the long‐term complications of these cells around these ZI hydrogels. Even though there are studies showing the long‐term presence of MNGCs around implants with no adverse effects on the surrounding tissue,^[^ ^49^ ^]^ these cells are generally considered an undesirable response as they can lead to chronic inflammation.^[^ ^50^ ^]^

Regarding cell infiltration into the implants, it has been previously shown that endogenous cells are able to infiltrate porous scaffolds in a porosity‐dependent manner.^[^ ^51^ ^]^ We also observed in this study that HA granular hydrogels were infiltrated by host cells throughout the whole implant with larger endogenous cell density for granular hydrogels with larger porosity (Figure 4C). It is also interesting to note that the relative densities of infiltrated cells into the HA granular hydrogels were higher than that of the surrounding tissue, especially for hydrogels with higher porosity (Figure 4D). However, the ZI granular hydrogels with both porosities did not support cell infiltration after 10 weeks. As both ZI and HA granular hydrogels made with small and large microgels had similar porosities and mechanical properties before implantation, the dramatically different in vivo cell infiltration behavior could be attributed to the distinct material and chemistries of these hydrogels. Zwitterionic hydrogels are generally known to be cell‐repellent.^[^ ^44^ ^]^ This could contribute to the inhibited cell infiltration into these granular hydrogels. Moreover, the dynamics and profile of porosity in vivo might be different for ZI and HA granular hydrogels due to different degradation behaviors, which could both contribute to and result from differential cell infiltration. As shown in Figure 2F, ZI granular hydrogels showed more swelling at longer timepoints in vitro, and even more so in the presence of collagenase (Figure S6, Supporting Information), resulting in reduced porosity. While this may suggest a similar trend in vivo, potentially leading to decreased cell infiltration, it should be noted that the in vivo environment is far more complex than the simplified in vitro conditions, which could result in different porosity changes. For example, early cell infiltration into granular hydrogels post‐implantation^[^ ^15^ ^]^ could significantly influence scaffold remodeling and alter the porosity profile over time. Therefore, it is essential to investigate hydrogel porosity changes and cell infiltration kinetics using labeled microgels at both early and later time points following in vivo implantation to better clarify this observation. Moreover, since to our knowledge, there are no other reports on in vivo implantation of zwitterionic granular hydrogels, more studies are required to investigate whether different architectures or compositions of zwitterionic hydrogels can affect cell infiltration.

### 2.4 Mechanical Fragmentation Dampens Collagen Capsule Formation Around Hydrogels

The in vivo performance of implants is often impeded by the formation of a dense, avascular, collagenous capsule around the implant which results in its isolation.^[^ ^52^ ^]^ This fibrotic collagen capsule compromises the diffusion of analytes, oxygen, and nutrients, contributing to implant failure.^[^ ^4^ ^]^ Many efforts have been made to mitigate FBR and fibrosis through material geometry or chemical modification.^[^ ^53^ ^]^ Super‐hydrophilic zwitterionic hydrogels have been shown to be particularly promising for solving this issue.^[^ ^28^ ^]^ Anti‐fibrotic effect of zwitterions is in principle due to their anti‐fouling properties that prevent the nonspecific protein adsorption on the implant surface which is considered the first, critical step in triggering capsule formation.^[^ ^54^ ^]^ Moreover, it has been shown previously that increased microporosity of the implants also results in reduced fibrosis and collagen capsule formation around implants.^[^ ^55^ ^]^

**Figure 5:**
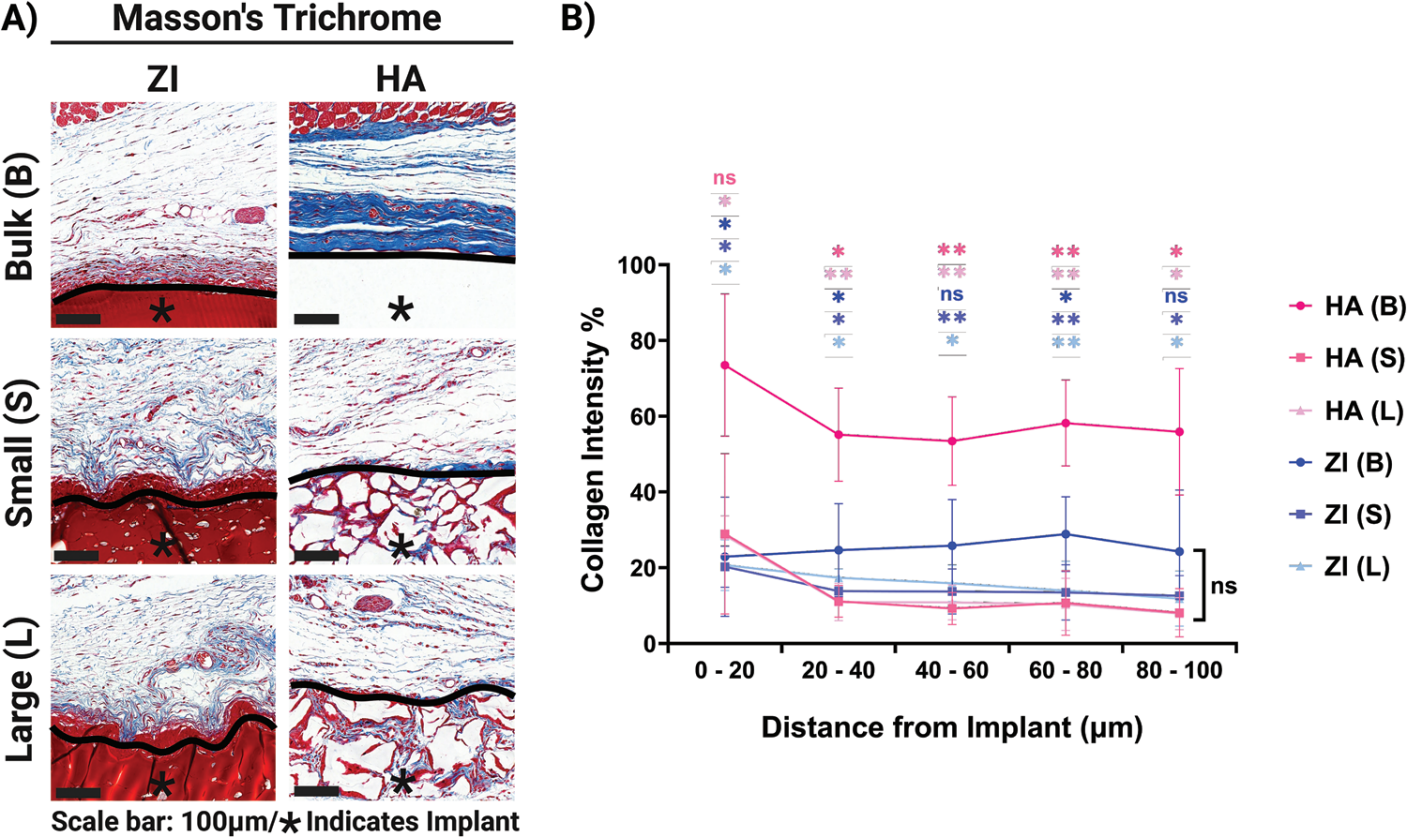
Collagen capsule formation around implants 10 weeks after subcutaneous implantation. A) Representative Masson's trichrome staining images. The blue staining indicates fibrotic collagen deposition surrounding implants (Scale bars: 100 µm; black line indicates hydrogel interface with animal tissue, asterisks (*) indicate the location of the implanted hydrogel). B) Quantification of collagen density around the implants up to 100 µm from the hydrogel interface. Data are represented as mean ± standard deviation. (*n* = 4 replicates).

We performed Masson Trichrome staining on samples to investigate fibrosis and collagen capsule density around implants (**Figure** **5**). We observed that both bulk and granular ZI hydrogels had a similarly low intensity of collagen staining around the implants, though, ZI granular hydrogels had even lower intensity compared to ZI bulk (Figure 5A). On the other hand, a dense and mature collagen capsule was observed around the HA bulk hydrogel, as evident with dark blue staining. Interestingly, both of the HA granular hydrogels had a significantly low intensity of collagen around them, similar to ZI granular hydrogels (Figure 5A). For hydrogels with similar microstructures, the biggest significant difference was observed for bulk hydrogels, where HA bulk had a dense capsule compared to ZI bulk (Figure 5B). All the granular hydrogels showed similar collagen intensities and there was no significant difference for different porosities or compositions of granular hydrogels (Figure 5B).

As we observed a large population of inflammatory cells and MNGCs around ZI implants (Figure 4) which are normally associated with FBR, the collagen capsule formation and maturation at a later timepoint around these implants needs to be studied. An important observation here is the significantly low collagen deposition for granular HA hydrogels compared to HA bulk. This highlights that not only material composition but also architecture plays an important role in fibrous capsule formation around implants. Similar results have been shown for silk‐based granular hydrogels made microfluidic setups.^[^ ^51a^ ^]^ As changing the material composition might not be possible for certain applications, increasing the porosity of the scaffold using granular counterparts of hydrogels could be a versatile method for dampening collagen capsule formation around implants.

### 2.5 Macrophage Population Around and Inside Granular Hydrogels is Controlled Both by Biomaterial Type and Porosity

**Figure 6:**
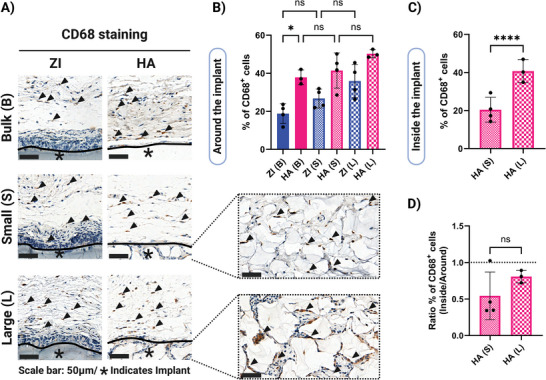
Macrophage population around and inside the implants 10 weeks after subcutaneous implantation. A) Representative CD68 immunostaining images (scale bars for all the images: 50 µm; black line indicates hydrogel interface with animal tissue, asterisks (*) indicate the location of the implanted hydrogel and black arrow indicates CD68^+^ macrophages). B) Quantification of percentages of CD68^+^ cells at the area adjacent to the implants and C) inside the implants. D) The ratio of CD68^+^ cells inside and around the HA granular hydrogels. Data are represented as mean ± standard deviation. Statistical significance was determined using a one‐way ANOVA with Tukey's multiple comparisons test (B,C) and unpaired *t*‐test (D). (non‐significant (ns) *p* > .05, ^∗^ *p* < .05, and ^∗∗∗∗^ *p* < .0001). (*n* = 4 replicates).

As monocytes and macrophages are amongst the first immune cells that come into contact with any implant and initiate the subsequent immune response after implantation,^[^ ^56^ ^]^ we stained our samples for a macrophage marker to identify the cell types surrounding and infiltrating the scaffolds. We performed immunostaining for the macrophage marker, CD68, which is known as a pan macrophage marker.^[^ ^57^ ^]^ As shown in **Figure** **6**A, CD68^+^ cells were found at the tissue interface of all the implants. ≈40% of cells surrounding HA bulk hydrogels were CD68^+^ macrophages, which was a significantly higher percentage than those surrounding ZI bulk hydrogels (Figure 6B). There were also similar percentages of macrophages around HA granular hydrogels with no significant difference for different porosities. The percentage of CD68^+^ cells was slightly less for ZI granular hydrogels compared to HA granular hydrogels, but this trend was not significant. Moreover, CD68^+^ cells were found to infiltrate into HA granular hydrogels, and the percentage of these cells was increased with increased porosity and microgel size with ≈20 and 40 percent for small and large microgels respectively (Figure 6C).

Percentages of CD68^+^ macrophages in the surrounding tissue versus those infiltrating the HA granular hydrogels were also compared. Interestingly, there were lower percentages of CD68^+^ cells inside the HA granular hydrogels compared to outside Figure 6D. It has been shown that preventing macrophage spreading by spatial confinement downregulates the pro‐inflammatory response of macrophages.^[^ ^58^ ^]^ This might be related to our findings showing lower percentages of CD68^+^ macrophages in the more confined environment inside the HA granular hydrogels compared to the surrounding tissue. However, we observed higher percentages of CD68^+^ macrophages for granular hydrogels with larger porosity and thus lower confinement. These results, taken together, show the importance of composition in macrophage recruitment to the implant interface, as well as the importance of porosity for the macrophage population inside the porous scaffolds. Future studies need to be conducted to further characterize the macrophage subtypes by examining different polarization states to clarify the immunomodulatory effects of the implant in vivo.

### 2.6 Mature Blood Vessels Grow Around Implants with Loose Collagen Capsules, and Inside Hyaluronic Acid Granular Hydrogels

**Figure 7:**
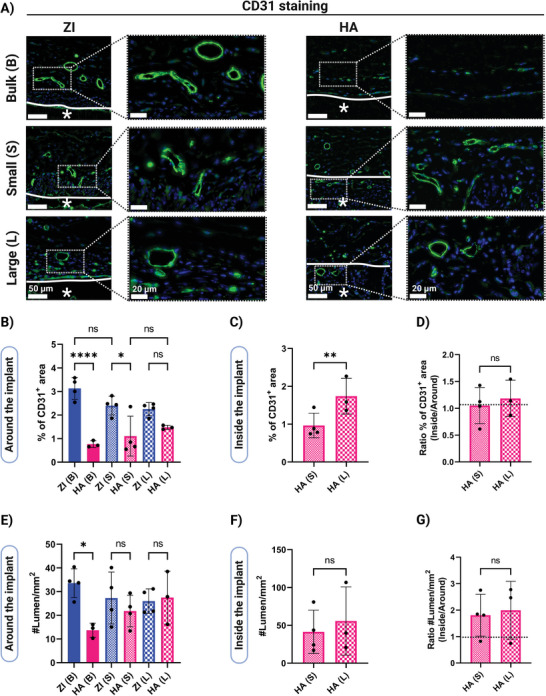
Vascularization around and inside implants 10 weeks after subcutaneous implantation. A) Representative CD31 immunostaining images of hydrogels (Scale bars: 50 µm and 20 µm from left to right zoom in images; white line indicates hydrogel interface with animal tissue and asterisks (*) indicate the location of the implanted hydrogel). B) Quantification of CD31^+^ cells at an area adjacent to the implants and C) inside the implants. D) The ratio of CD31^+^ cells inside and around the HA granular hydrogels E) Quantification of the number of lumens per area at an area adjacent to the implants and F) inside the implants. G) The ratio of the number of lumens per area inside and around the HA granular hydrogels. Data are represented as mean ± standard deviation. Statistical significance was determined using a one‐way ANOVA with Tukey's multiple comparisons test (B, C, E, and F) and unpaired *t*‐test (D and G). (non‐significant (ns) *p* > .05, ^∗^ *p* < .05, ^∗∗^ *p* < .01, and ^∗∗∗∗^ *p* < .0001). (*n* = 4 replicates).

Angiogenesis and vessel ingrowth are important factors for biomaterial integration and endogenous tissue repair. It has been shown that zwitterionic hydrogels promote neovascularization around the implants after in vivo implantation.^[^ ^28b^ ^]^ Also, porous scaffolds have been shown to promote vessel invasion and angiogenesis in vivo.^[^ ^9^, ^55^, ^59^ ^]^ We evaluated angiogenesis around and within the scaffolds using immunostaining for an endothelial cell marker CD31 (**Figure** **7**). As shown in Figure 7A, CD31^+^ cells were observed around all the samples. However, mature luminal structures were seen only around the ZI bulk and all the ZI and HA granular hydrogels. This is in line with the trend observed in fibrous capsule formation around samples. HA bulk hydrogel was the sample with a mature and dense collagen capsule and also is the one with the lowest vasculature formation around it. It has been previously shown that a dense collagen capsule around the implant has low vascular density.^[^ ^28^, ^60^ ^]^

Quantification of the CD31^+^ area around the implants shows in general a larger area for ZI hydrogels compared to HA hydrogels with the most striking difference observed between ZI bulk and HA bulk hydrogels (Figure 7B). However, the CD31^+^ area for different porosities of the same biomaterial is not significantly different. CD31^+^ endothelial cells were also observed inside the HA granular hydrogels. As shown in Figure 7C, we observed that the CD31^+^ area inside the HA granular hydrogel with larger porosity was significantly higher. We also compared the CD31^+^ area inside and around the HA granular hydrogels (Figure 7D), showing a similar extent of CD31^+^ endothelial cell invasion inside the HA granular hydrogels to the surrounding tissue.

We also investigated blood vessel formation by quantifying the density and diameters of luminal structures. The average individual lumen areas as well as the density of these structures reported as #lumen/mm^2^ were analyzed. As shown in Figure S11A (Supporting Information), large blood vessels with areas of more than 1000 µm^2^ were observed around all scaffolds, except for HA bulk. As shown in Figure 7E, the density of these lumens around implants was similar for all samples except for HA bulk, having significantly less luminal structures. Blood vessels were also observed within HA granular hydrogels (Figure 7A). The number of luminal structures per area was slightly higher for HA granular hydrogels with higher porosity but not significantly different (Figure 7F). Also, there was no significant difference in average area of individual lumens for the two different porosities of HA granular hydrogels (Figure S11B, Supporting Information). Moreover, blood vessels at the implant‐host‐tissue interface were further stained with α‐smooth muscle actin (α‐SMA), as shown in Figure S12 (Supporting Information).

Interestingly, when comparing lumen density inside and around HA granular hydrogels, the number of luminal structures inside these hydrogels was higher than that in the adjacent tissue, showing that these granular hydrogel structures are able to promote vasculature formation (Figure 7G). Also, the average of individual lumens area was not significantly different for the vasculature around compared to inside the granular hydrogels for both porosities (Figure S11C, Supporting Information), showing that the pore size within these scaffolds was not a limiting factor for lumen maturation. Studies have shown that high molecular weight HA in its native form inhibits angiogenesis, while short‐chain HA and HA degradation products are pro‐angiogenic.^[^ ^61^ ^]^ Thus, we hypothesize that the increased area of fragmented HA on the surface of the HA microgels, and possible degradation products, might contribute to this enhanced angiogenesis and vasculature formation inside the HA granular hydrogels. To our knowledge, this is the first report on mature blood vessel formation inside granular hydrogels made with mechanical fragmentation, showing the versatility of this method in producing hydrogels for endogenous tissue repair.

## 3 Discussion

Granular hydrogels are an emerging biomaterial class with strong potential for diverse tissue engineering applications both as tissue models in vitro and as injectable or implantable scaffolds for in vivo tissue repair. This study provides new insights on in vitro immune response and long‐term in vivo stability and fibrotic response to granular hydrogels with varied biomaterial (ZI vs HA) and porosity, fabricated by mechanical fragmentation and a tunable secondary enzymatic annealing.

Our in vitro results showed an overall stronger inflammatory response of HA granular hydrogels compared to the non‐stimulatory effect of ZI ones, which is in line with previous literature. It has been shown in several studies that zwitterionic hydrogels have immunomodulatory effects on macrophages in vitro.^[^ ^28^, ^62^ ^]^ For example, macrophages seeded onto zwitterionic hydrogels show inhibition of inflammatory pathways activation in response to LPS and Interferon‐gamma and exhibit a more anti‐inflammatory phenotype.^[^ ^28^, ^62^ ^]^ Similar results were observed for alginate microcapsules modified with zwitterionic polymers compared to non‐modified capsules.^[^ ^62b^ ^]^ It has been also shown that zwitterionic functionalization of biomaterials significantly decreases their phagocytosis by THP1 cells.^[^ ^63^ ^]^ On the other hand, it has been shown that short‐chain HA molecules in soluble form have pro‐inflammatory effects.^[^ ^64^ ^]^ Moreover, fragmented HA molecules can act as damage‐associated molecular patterns (DAMPs) and promote the activation of immune response.^[^ ^65^ ^]^ Thus, we hypothesize that the increased surface area of fragmented HA chains in HA granular hydrogels might contribute to the observed increased immune response of these hydrogels compared to bulk.

Our in vivo results, being the first long‐term (10 week) investigation of granular hydrogels in an immunocompetent animal model, demonstrated reduced collagen capsule formation around granular hydrogels compared to bulk and differential cell infiltration behaviors based on the constituting material. Zwitterionic granular hydrogels remained acellular and were surrounded by numerous host cells including MNGCs as well as mature blood vessels. Several studies have suggested giant cells as the main drivers of neovessel formation,^[^ ^46^ ^]^ which correlates with our observations. In contrast, HA granular hydrogels exhibited fewer surrounding cells, with more mature blood vessels in the pores between microgels and an increased macrophage population in a porosity‐dependent manner. Similarly, a higher CD31+ endothelial cell population in the implants has been previously shown to strongly correlate with an increased presence of CD68+ macrophages.^[^ ^66^ ^]^

Regarding the correlation between in vitro and in vivo immunomodulation studies, increased stimulatory response for HA hydrogels in vitro, is in line with a higher percentage of CD68^+^ macrophages observed for these scaffolds after in vivo implantation. However, it is important to note that even though zwitterionic hydrogels did not support macrophage adhesion and stimulation in the in vitro setting, many host cells and MNGCs were observed surrounding these hydrogels in vivo. This result is in contrast with a significant body of current literature on the biocompatibility of zwitterionic hydrogels in vivo.^[^ ^28^ ^]^ At the same time, there are studies that report MNGC formation and inflammatory responses to zwitterion‐containing hydrogels, indicating the need for careful formulation.^[^ ^43^, ^67^ ^]^ Notably, factors such as implant size and architecture have been shown to greatly influence immune response in vivo.^[^ ^68^ ^]^ Thus, the mechanically fragmented particles, which have irregular surfaces, might play an important role in this response. Further studies comparing these scaffolds with other smoother surfaces may provide deeper insights. Moreover, the varied in vitro and in vivo results for zwitterionic granular hydrogels could arise from the interplay between composition, implant shape, and degradation behavior within the complex in vivo environment, which involves interactions with multiple cell types, as opposed to the isolated single‐cell‐type interactions typically studied in vitro. Another important factor is the duration of the study. While the in vivo results reflect a long‐term, 10‐week process, in vitro studies often focus on short‐term interactions (24 h). These results taken together indicate that there should be extra caution in drawing conclusions and predictions on in vivo responses to biomaterials solely based on in vitro results. Moreover, while replicating the complex in vivo environment in an in vitro setting is challenging, using multicellular systems and extending time points in vitro could provide more predictive insights for future in vivo studies.

## 4 Conclusion

In this work, we fabricated granular hydrogels based on ZI and HA using mechanical fragmentation. For each material, we produced two sets of granular hydrogels with different porosities, ≈14 and 20%, to investigate the effect of both porosity and composition on in vitro and in vivo immune responses. Our in vitro studies showed that mechanical fragmentation of ZI hydrogels has no effect on their stimulatory properties as both bulk and granular ZI hydrogels were non stimulatory. However, fragmented, granular HA hydrogels activated inflammatory pathways in vitro, compared to the starting bulk hydrogel. Our in vivo results showed that HA granular hydrogels inhibit fibrotic encapsulation and promote tissue ingrowth and vascularization compared to HA bulk hydrogels. Conversely, we observed that the ZI granular hydrogels were not infiltrated by host cells and instead were surrounded by large numbers of cells including macrophages, MNGCs, and endothelial cells in the biomaterial‐host tissue interface. Overall, our results show two distinct immune responses toward the two types of granular hydrogels, suggesting different future directions and applications. HA granular hydrogels, having enhanced cell and vasculature ingrowth, show promise for tissue repair applications. In contrast, ZI granular hydrogels, resisting cell infiltration, could potentially be used as carriers or tissue fillers in situations where host cell invasion is undesirable and should be minimized. However, further characterization and optimization are needed, particularly concerning the accumulation of MNGCs, to fully explore their potential.

As we have previously shown that these ZI granular hydrogels support the viability of encapsulated cells,^[^ ^29^ ^]^ it would be interesting to investigate how cell‐laden ZI granular hydrogels will perform in vivo. Specifically, as it has been shown that macrophage invasion into cell‐laden HA granular hydrogels can be detrimental and lead to degradation of in vitro engineered tissue,^[^ ^22^ ^]^ it would be interesting to investigate how cell‐laden ZI granular hydrogels or a combinatory scaffold made of both HA and ZI will perform in this context. Also, further studies are needed to look at the dynamics of porosity changes and immune response at different times after implantation. In this study, we only looked at a relatively long timepoint to analyze scaffold stability and fibrotic response, while earlier timepoints to investigate acute inflammatory response as well as much later timepoints to study long‐term compatibility and integration are required.

## 5 Experimental Section

### Materials

Hyaluronic acid (HA) (intrinsic viscosity: 1.92 m3 kg^−1^) was purchased from HTL Biotechnology. Gelatin from porcine skin Type A and lipopolysaccharides (LPS) from Escherichia coli O127:B8 and, were purchased from Sigma. RPMI‐1640 medium, penicillin‐streptomycin, GlutaMAX, MEM non‐essential amino acids solution, sodium pyruvate, and fetal bovine serum (FBS) were obtained from Gibco. All other solvents and chemicals were purchased from Sigma.

### Carboxybetaine Acrylamide (CBAA) Synthesis

The monomer was synthesized using a previously reported protocol,^[^ ^29^ ^]^ with minor modifications. In a 100 mL flask, DMAPA (8.9 g, 57.28 mmol, 1 eq) was dissolved in 60 mL anhydrous THF and the flask was sealed with a dropping funnel and placed to −10 °C ethanol bath. Beta‐Propiolactone (5 mL, 79.86 mmol, 1.4 eq) dissolved in 15 mL anhydrous THF was added to the dropping funnel and dropped into the solution under stirring. The reaction mixture was allowed to warm to room temperature overnight and the resulting white suspension was placed in the freezer for another 24 hrs at −20 °C to precipitate the product. Finally, the mixture was filtered, washed with dry diethyl ether, and the product was obtained with a vacuum filter, washed several times with cold ether, and dried overnight under high vacuum.

### Tyramine Acrylamide (TyrAA) Synthesis

The monomer was synthesized using a previously reported protocol.^[^ ^29^ ^]^ In a 100 mL flask, tyramine hydrochloride (2.0 g, 11.52 mmol, 1 eq) was dissolved in 32 mL DMF, and DIPEA (6 mL, 34.55 mmol, 3 eq) was added. The solution was degassed with nitrogen for 15 min and cooled in 0 °C ice bath. Acryloyl chloride (1.2 mL, 14.97 mmol, 1.3 eq) dissolved in 3 mL DMF was slowly dropped into the solution under vigorous stirring. The reaction mixture was allowed to warm to room temperature and stir overnight. The solvent was removed on a rotary evaporator and the precipitate was redissolved in 10 mL ethyl acetate and then transferred to −20 °C to allow for crystallization overnight. The product was obtained with a vacuum filter, washed with cold chloroform, and dried overnight under vacuum.

### Gelatin Methacrylate (GelMA) Synthesis

GelMA was synthesized as previously described.^[^ ^18^ ^]^ The degree of substitution (DS) was estimated with ^1^H NMR (Bruker Ultra shield 400 MHz) in D_2_O. GelMA lysine integration signal (2.95–3.05 ppm) was compared to unmodified gelatin lysine integration signal (2.95–3.05 ppm). Phenylalanine signal (7.2–7.5 ppm) was used as an internal reference. DS was found to be 90%.

### Hyaluronic Acid Tyramine (HA‐Tyr) Synthesis

HA‐Tyr was synthesized using a previously reported protocol.^[^ ^69^ ^]^ The degree of substitution (DS), defined as the number of substituted groups per 100 disaccharide units, was calculated from ^1^H NMR (Bruker Ultra shield 400 MHz) in D_2_O by comparing the integral values of the aromatic protons of tyramine (peaks at 6.86 and 7.17 ppm) and the methyl protons of HA (1.9 ppm). DS was found to be 10%.

### Zwitterionic Bulk Hydrogel Formation

Bulk zwitterionic hydrogel was produced by photopolymerization of CBAA (2.5 m) and TyrAA (0.125 m) using GelMA (0.002 M, ≈1.5 weight fraction) as the crosslinker and LAP (0.05 wt%) as photoinitiator. The precursor solution was prepared by dissolving all the components in Milli‐Q water in a 37 °C water bath. The solution was degassed with nitrogen for 10 min and then injected between two glass slides with a 1 mm polytetrafluoroethylene spacer. Photopolymerization was initiated by UV–vis (405 nm, 30 min), and the resulting hydrogels were dialyzed in deionized (DI) water for at least 5 days to remove unreacted monomers and reagents. For sterile bulk hydrogel preparation, the precursor solution was sterile filtered under a laminar hood, the molds were sterilized with UV, and the resulting hydrogel was dialyzed against sterile saline (0.9 wt% NaCl) solution.

### Hyaluronic Acid Bulk Hydrogel Formation

Bulk hyaluronic acid hydrogel was prepared by enzymatic crosslinking of HA‐Tyr (2 wt%) using HRP (0.000625 wt% – 0.93 U mL^−1^) and H_2_O_2_ (0.005%). The precursor solution was prepared by dissolving HA‐Tyr as 2 wt% in saline (0.9 wt% NaCl) overnight and then mixing with HRP. The solution was cast in cylindrical PDMS molds and gelation was initiated by adding H_2_O_2_. For sterile bulk hydrogel preparation, the precursor solution was sterile filtered under a laminar hood, the molds were sterilized with UV, and the resulting hydrogel was dialyzed against sterile saline (0.9 wt% NaCl) solution.

### Bulk Hydrogel Equilibrium Water Content (EWC) Measurement

After dialysis, the swollen bulk hydrogels were weighed and freeze‐dried, and the dried hydrogels were weighed again. EWC was calculated as the ratio of water mass (swollen hydrogel weight minus dried hydrogel weight) to the swollen hydrogel mass.

### Microgels and Granular Hydrogel Preparation

Microgels were prepared by mechanical fragmentation of the bulk hydrogels. Equilibrated hydrogels were cut into small pieces and transferred into a 10 mL custom‐made extruder connected to a metal sieve with a mesh width of 150 or 50 µm. For 150 µm‐grid microgels, bulk gels were manually sieved three consecutive times with the 150 µm mesh, and for 50 µm‐grid microgels, the resulting microgels were additionally sieved another three times with the 50 µm mesh. Microgels were then sterilized by precipitation in ethanol, dried overnight in a vacuum oven, re‐suspended in sterile water, and lyophilized. To make granular hydrogels, the lyophilized microgels were first resuspended in sterile saline (0.9 wt% NaCl) solution as 5.5 wt% for zwitterionic microgel and 3.5 wt% for hyaluronic acid microgels, matching their EWC. To prepare 200 µL of granular hydrogel, 180 µL of microgels were mixed with 10 µL of HRP (0.2 wt%, 300 U mL^−1^) and 10 µL of H_2_O_2_ (0.1%) in cylindrical PDMS molds and incubated for 30 min.

### Microgel Diameter and Granular Hydrogel Porosity, Pore Diameter and Tortuosity Measurement

For zwitterionic materials, labeled microgels were prepared by adding fluorescein o‐acrylate comonomer to the bulk hydrogel precursor solution at a final concentration of 0.018 wt%. For hyaluronic acid, tetramethylrhodaminisothiocyanat‐Dextran (TRITC‐Dextran) was added to the bulk hydrogel precursor solution at a final concentration of 0.1 wt%. Labeled microgel suspension was prepared at a low concentration of ≈0.5% to facilitate microgel separation under the microscope. Microgels were dispersed onto glass slides and imaged using a fluorescent microscope (SP8, Leica). The microgel area was determined, and the diameter was calculated as the equivalent diameter of a circle with the same area using ImageJ software's particle analysis tool. For porosity and pore diameter measurements, annealed granular hydrogels, prepared as described previously, were imaged using confocal microscopy (SP8, Leica). The void fraction was determined by converting the image stacks into single images and applying a threshold to identify the void spaces. Cross‐sectional areas occupied by voids were calculated for each image and averaged across the entire stack to determine porosity. Pore diameter was evaluated similarly to microgel diameter, based on pore area. Blender 2.91 simulations were used to calculate interconnectivity and path tortuosity, using the Bullet Physics engine. Hydrogel structures were reconstructed using Imaris software and subsequently imported into Blender for further processing. Spherical beads (diameter = 0.2 units) with active rigid bodies were dropped into the hydrogel reconstructions (scaled to 0.02) using spherical collision shapes. A total of 100 beads, arranged in a plane, were placed directly above the hydrogels (z = 0) and dropped under the influence of gravity. Each simulation consisted of 250 frames. The xyz coordinates of each bead's position were extracted at every frame. Tortuosity was then quantified by dividing the trajectory length (calculated from the extracted position data) by the scaffold thickness.

### SEM Imaging

Bulk and granular hydrogels were first washed with Milli‐Q water and then gradually chemically dried through a dehydration process. The dehydration steps started by incubating the samples in a series of ethanol solutions with increasing concentrations (30, 50, 70, 90, and 100%), each for 1 h. Following ethanol dehydration, the samples were treated by hexamethyldisilazane (HMDS) in ethanol at the same concentrations (30, 50, 70, 90, and 100%), again for 1 h each. After the final 100% HMDS incubation, the samples were left to dry in a fume hood. Once fully dried, a 10 nm carbon layer was applied using a CCU‐010 Carbon Coater (Safematic), and imaging was conducted with a Zeiss Merlin scanning electron microscope (SEM) at an operating voltage of 2 kV.

### Compression Testing

Unconfined compression experiments were performed on a TA.XTplus texture Analyzer (Anton Paar) equipped with a 500 g load cell. For each sample (6 mm in diameter and 2 mm in height), a pre‐load was applied to the sample until it reached full contact with the plate and was then allowed to relax completely. Samples were compressed at a rate of 0.01 mm s^−1^ until they reached 15% strain. The compressive modulus was extracted from the slope of the first linear part of the stress versus strain curve.

### Swelling and Enzymatic Degradation

For swelling analysis, 6 mm hydrogel disks were prepared as described in the previous sections, weighed, and transferred to wells of 24‐well plate containing 2 mL 1 × PBS, and incubated at 37 °C. At regular intervals, supernatants were removed, and the samples were weighed. The swelling ratio was determined as the ratio of hydrogel mass at a given time point divided by its initial mass and reported as a percentage. For degradation analysis, collagenase as 20 U mL^−1^ or Hyaluronidase as 10 U mL^−1^ in PBS or was used and the enzyme solution was refreshed every day to ensure continuous enzyme activity.

### THP‐1 Cell Culture

THP‐1 dual cell line (Cat. thpd.nfis) was purchased from InvivoGen, USA. Cells were cultured in suspension in T‐150 cm^2^ cell culture flask in RPMI‐1640 medium supplemented with 10% FBS, 1% penicillin‐streptomycin, 1% GlutaMAX 100‐X, 1% MEM Non‐Essential Amino Acids Solution 100‐X and 1% sodium pyruvate at 37 °C, 5% CO_2_ and 95% humidity. Suspended cells were split by centrifugation and the cell pellet was resuspended in fresh medium. Cells were cultured on hydrogels (6 mm in diameter and 2 mm in height) at a density of 2.5 × 10^5^ cells mL^−1^ without or with 100 ng mL^−1^ LPS for 24 hrs. Afterward, the supernatant was analyzed for NF‐κB and IRF pathway activation, and the cells were harvested for gene expression analysis by qRT‐PCR. THP‐1 dual cells have a stable integrator‐secreted alkaline phosphatase (SEAP) reporter gene for monitoring NF‐κB and IRF activation. Both reporter proteins were readily measurable in the cell culture supernatant using QUANTI‐Blue, a SEAP detection reagent, and QUANTI‐Luc, a Lucia luciferase detection reagent, according to the manufacturer's instructions.

### THP‐1 Differentiation

To differentiate THP‐1 cells into adherent and nonpolarized M0 macrophages, cells were seeded as 1 × 10^5^ cells cm^−2^ in well plates and incubated with RPMI‐1640 medium supplemented with 5 ng mL^−1^ phorbol myristate acetate (PMA) for 24 h. Afterward, the medium was aspirated, and the adherent macrophages were washed twice with PBS and cultured for 48 h in a PMA‐free medium. Next, the adherent M0 macrophages were detached by 0.25% trypsin/0.02% EDTA solution (Merck), and 2 × 10^5^ cells were seeded on hydrogels (6 mm in diameter and 2 mm in height) or ctrl polystyrene surfaces for 24 h. Afterward, the supernatant was analyzed for NF‐κB and IRF pathway activation, and the cells were harvested for gene expression analysis by qRT‐PCR. For the cell adhesion experiment, samples were washed several times with medium, 24 h after the seeding, and stained with DAPI for cell nuclei, and then imaged with a microscope. The number of cells per area was analyzed by ImageJ.

### RNA Isolation and qRT‐PCR Analysis

Gene expression levels were assessed through quantitative real‐time PCR (qRT‐PCR) analysis. Extraction of RNA was performed with NucleoZol (MachereyNagel) according to the manufacturer's instructions. Retreotranscription to cDNA was performed with GoScript Reverse Transcriptase (Promega) and cDNA was subsequently diluted 1:5 with RNAse‐free water for q‐PCR analysis. The analyzed genes were IL‐6 (Fw: GAAAGCAGCAAAGAGGCACT, Rv: TTTCACCAGGCAAGTCTCCT) and IL‐1β (Fw: CTGAGCTCGCCAGTGAAATG, Rv: TCTGTTTAGGGCCATCAGCTT). The qRT‐PCR was performed with GoTaq qPCR Master Mix (Promega) on a QuantStudio 3 device (Applied Biosystems).

### In Vivo Hydrogel Implantation and Retrieval

The animal study was performed in compliance with the ethical license (Application No. ZH024/2022). 12‐week‐old, male and female, C57BL/6 mice were obtained from Janvier Labs. Animals were allowed to acclimatize to the new housing environment for 2 weeks before the beginning of the experiments. Mice were housed in groups of 3 in IVC cages (Green Line, Tecniplast) at SOPF hygiene. Samples were randomly allocated to the animals (N = 4, 2 females and 2 males); one sample per animal. Anesthesia was induced with 5% isoflurane following the maintenance of anesthesia during the procedure with 1.5‐3% isoflurane. Before the surgery, all animals received an initial analgesia treatment of Meloxicam (Metacam, s.c. 2 mg kg^−1^), and eye cream (Bepanthen) was applied to prevent corneal dissection. The animals were shaved on their backs and the skin was cleaned with an alcohol‐based disinfectant (Kodan forte). A minimal‐sized incision was made dorsally, slightly to one side; afterward, a subcutaneous pocket was opened toward the lateral side using a blunt tool. The equilibrated hydrogel disks (10 mm in diameter and 3 mm in height) were implanted into the pocket. The incisions were closed with surgical staples (3M) and a spray bandage (Opsite) was applied. The staples were removed after 1 week. After 10 weeks, animals were euthanized by CO_2_ asphyxiation. The retrieved hydrogel disks together with surrounding tissues were dissected and fixed in a 4% phosphate‐buffered formalin solution.

### Histology and Immunohistochemistry

Fixed samples were dehydrated in an ethanol sequence, embedded in paraffin wax (Milestone LogosJ), and cut into 5‐µm sections on a microtome. Samples were progressively deparaffinized and rehydrated before staining. Brightfield images of stained sections were recorded on a 3DHistech Pannoramic 250‐slide scanner and visualized with the case viewer 2.4 software.

### Hematoxylin and Eosin (H&E) Staining

The cellularity around and inside samples was examined by staining with H&E, in which cell nuclei appear dark purple and cell cytoplasm appear pink. Samples were stained in Mayer's Hematoxylin for 8 min, rinsed under tape water for 10 min, and then counterstained with Eosin Y for 1 min. Counting of cell density inside and around the implants (500 × 300 µm: 500 µm parallel to the implant surface and 300 µm from implant interface into the surrounding tissue) was performed using Image J after color deconvolution to isolate and count cell nuclei. For the area around the implant, 3 images from 3 different areas per sample were randomly selected, analyzed, and then averaged.

### Masson's Trichrome Staining

The collagen capsule around samples was examined by Masson's Trichrome staining, in which collagen fibers appear blue, muscle fibers red, and cell nuclei appear black. Samples were stained in pre‐heated Bouin's fluid for 60 min, Weigert's Hematoxylin for 5 min, Biebrich Scarlet/Acid Fuchsin for 15 min, and then Phosphomolybdic/Phosphotungstic Acid for 15 min with rinsing in tap water in between. Then, without rinsing, samples were stained in Aniline blue for 10 min, rinsed, and then acetic acid 1% was applied for 1 min. The blue‐pixel density was measured using Image J software after color deconvolution. Random images were taken from the surrounding tissue starting from the interface with the hydrogel sample. The intensity along a straight line from hydrogel sample up to 100 µm was measured and reported as collagen intensity. 3 lines per image and 3 images per sample were analyzed and averaged.

### CD68 Staining

Macrophage population around and inside samples were analyzed using CD68 staining. Heat‐induced epitope retrieval was performed on slides with boiling sodium citrate buffer for 20 min. Sections were blocked with 5% goat serum, 1% BSA, and 0.1% Triton for 1 h at room temperature. The primary antibody, rabbit anti CD68 (1:200, ab125212, Abcam), dissolved in 1% BSA in PBS was applied and sections were incubated overnight at 4 °C. Sections were incubated with the secondary antibody, goat anti‐rabbit IgG‐HRP (1:1000, ab6721, Abcam), in 1% BSA in PBS for 1 h and developed with the DAB substrate kit (ab64238, Abcam) for 5 min. Sections were stained with Weigert's iron hematoxylin (Thermo Fisher Scientific) for 3 min, destained in 1% acid‐alcohol, and blued in 0.1% Na2CO3. Counting of CD68 positive cells was performed using Image J after color deconvolution to isolate nuclei (blue) and positive cells (brown).

### CD31 Staining

Vascularization around and inside samples were analyzed using CD31 staining. Heat‐induced epitope retrieval was performed on slides with boiling sodium citrate buffer for 20 min. Sections were blocked with 5% BSA, and 0.1% Triton for 1 h at room temperature. The primary antibody, goat anti CD31 (1:200, AF3628‐SP, R&D systems), dissolved in 1% BSA in PBS was applied and sections were incubated overnight at 4 °C. Sections were incubated with the secondary antibody, Alexa Flour 488 donkey anti‐goat (1:500, A‐11055, Invitrogen), in 1% BSA in PBS for 1 h. Cell nuclei were stained with DAPI (1:1000). Counting of CD31 positive area and luminal structures was performed using Image J.

### α‐SMA Staining

Heat‐induced epitope retrieval was performed on slides with boiling sodium citrate buffer for 20 min. Sections were blocked with 5% BSA, and 0.1% Triton for 1 h at room temperature. Primary mouse Alpha‐Smooth Muscle Actin (α‐SMA) Monoclonal antibody (1:500, 14‐9760‐82, Invitrogen), dissolved in 1% BSA in PBS was applied and sections were incubated overnight at 4 °C. Sections were incubated with the secondary antibody, Alexa Flour 488 goat anti‐mouse (1:1000, A32723, Invitrogen), in 1% BSA in PBS for 1 h. Cell nuclei were stained with DAPI (1:1000).

### Statistical Analyses

All data were presented with individual data points on the graphs and bar plots with error bars represent mean ± standard deviation with n ≥ 3. Statistical analyses were performed in GraphPad Prism 9.2.0 software. An unpaired *t* test or one‐way or two‐way ANOVA with Tukey's multi‐comparison test was used to analyze the data. A level of *p* < 0.05 was considered significant. The p‐values for statistical significance were represented with stars (^∗^ *p* < .05, ^∗∗^ *p* < .01, ^∗∗∗^ *p* < .001, and ^∗∗∗∗^ *p* < .0001).

## Conflict of Interest

The authors declare no conflict of interest.

## Supporting information



Supporting Information

## Data Availability

The data that support the findings of this study are openly available in ETH Research Collection at https://doi.org/10.3929/ethz‐b‐000638135, reference number 638135.
