# Supplementary material for: Foreign Body Immune Response to Zwitterionic and Hyaluronic Acid Granular Hydrogels Made with Mechanical Fragmentation
Source: Adv Healthc Mater. 2024 Nov 5;14(2):2402890. doi: 10.1002/adhm.202402890 (PMC11730820; doi:10.1002/adhm.202402890)
Supplement: Supplementary file 1 — Supporting Information [file ADHM-14-0-s001.pdf]

ADVANCED HEALTHCARE MATERIALS

Supporting Information

for *Adv. Healthcare Mater.*, DOI 10.1002/adhm.202402890

Foreign Body Immune Response to Zwitterionic and Hyaluronic Acid Granular Hydrogels Made with Mechanical Fragmentation

*Maryam Asadikorayem, Patrick Weber, František Surman, Anna Puiggalí-Jou and Marcy Zenobi-Wong**

Supporting Information

Foreign body immune response to zwitterionic and hyaluronic acid granular hydrogels made with mechanical fragmentation

*Maryam Asadikorayem, Patrick Weber, František Surman, Anna Puiggalí-Jou, Marcy Zenobi-Wong**

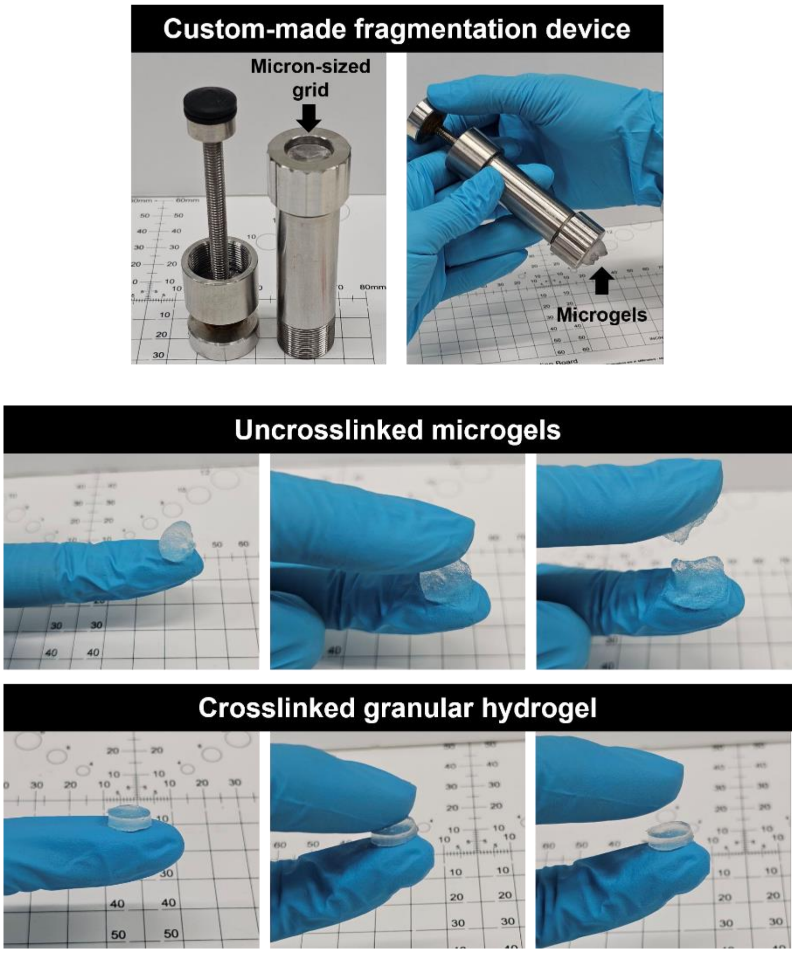

Figure S1. Mechanical fragmentation device and fragmented microgels shape retention and stability before and after secondary enzymatic crosslinking.

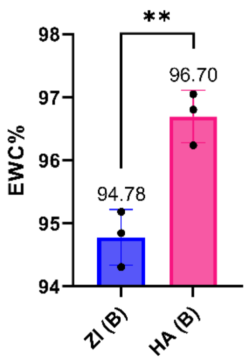

Figure S2. Equilibrium water content (EWC) of the bulk zwitterionic (ZI) and hyaluronic acid (HA) hydrogels.

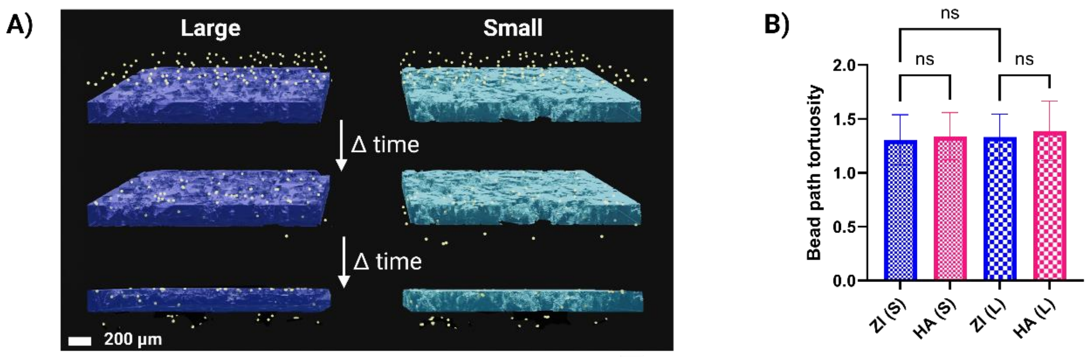

Figure S3. **A)** Simulations with Blender for ZI granular hydrogels made with small microgels and large microgels at three different time frames (0, 15 and 45) showing pore interconnectivity. (Scale bar 100 μm. **B)** Bead path tortuosity.

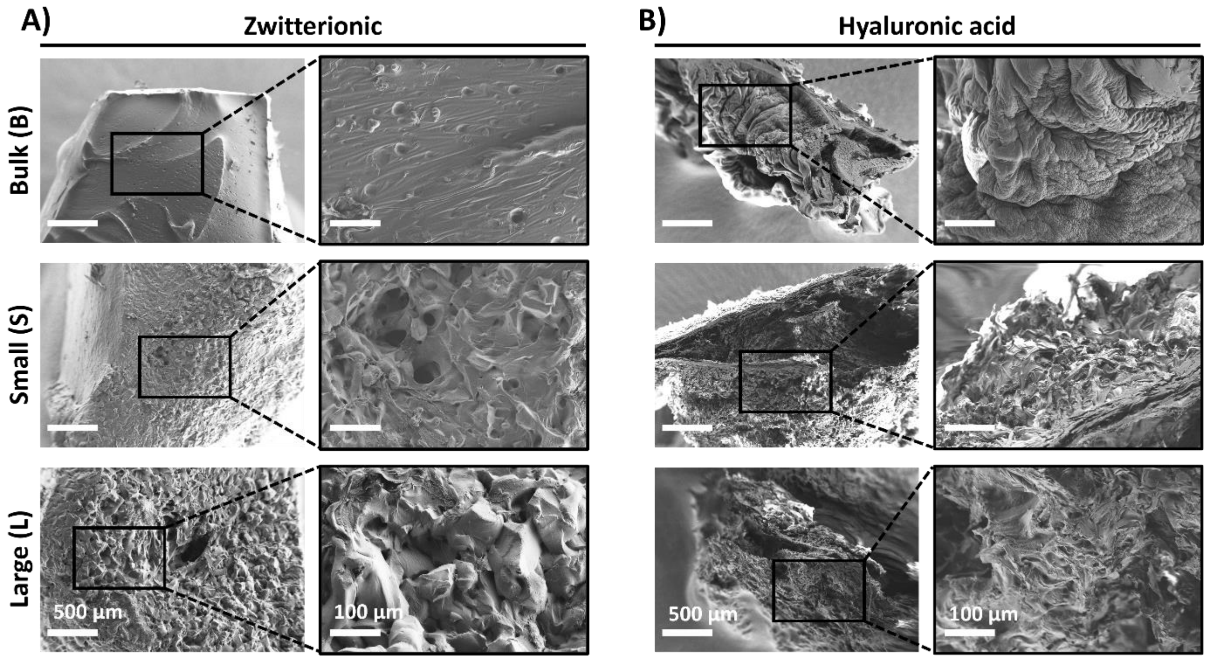

Figure S4. Representative SEM micrographs showing the cross section of **A)** zwitterionic and **B)** hyaluronic acid bulk and granular hydrogels. (Scale bar: 500 µm, insert 100 µm).

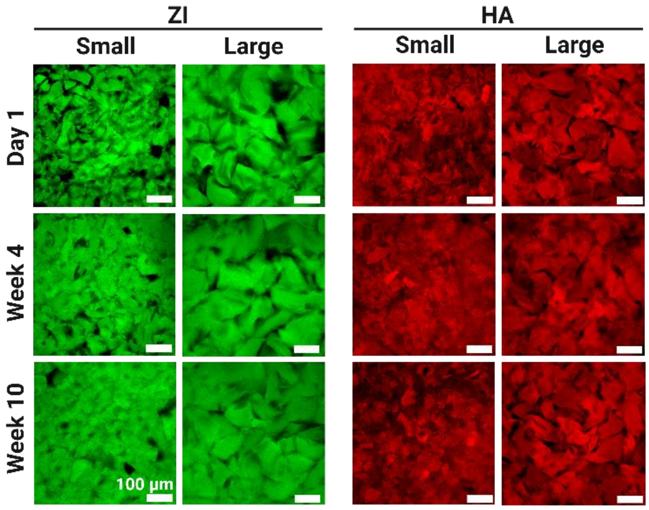

Figure S5. Confocal images of labeled ZI and HA granular hydrogels over 10 weeks of incubation in PBS (Scale bar 100 µm).

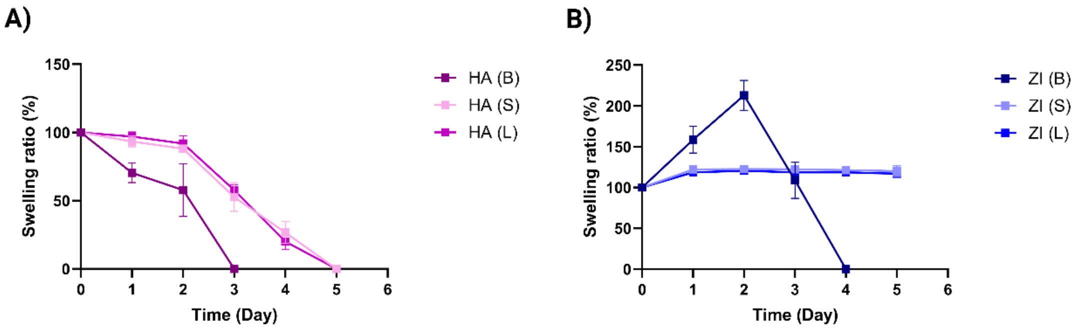

Figure S6. The swelling ratio of bulk and granular hydrogels incubated with either **A)** Hyaluronidase (10 U mL^{-1}) for HA or **B)** collagenase (20 U mL^{-1}) for ZI hydrogels over 6 days. (n=3)

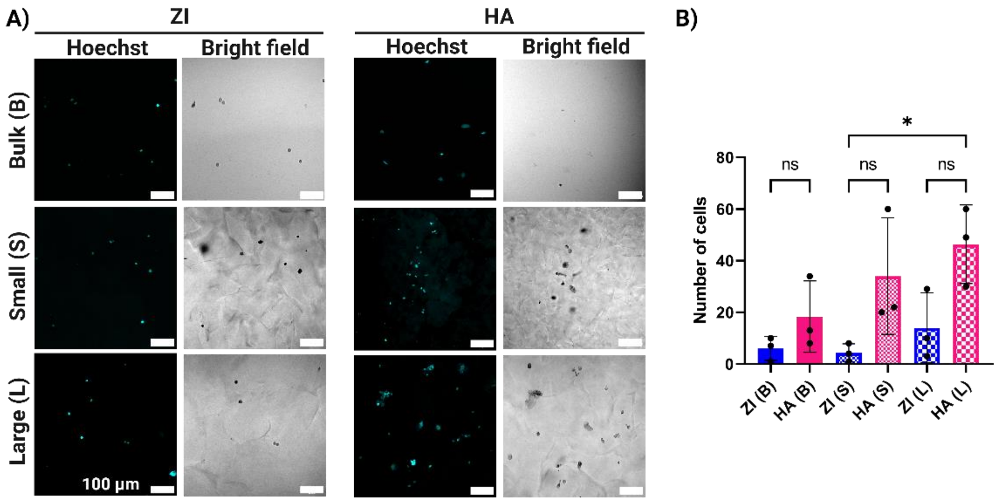

Figure S7. *In vitro* cell adhesion with THP-1 derived macrophages. A) Confocal and bright field images of macrophages attached to hydrogels after 24 hrs of seeding (Scale bar 100 µm). **B)** Quantification of number of attached cells per area. Data are represented as mean ± standard deviation. Statistical significance was determined using a one-way ANOVA with a Tukey's multiple comparisons test (non-significant (ns) p> .05 and ∗p < .05. (n = 3 replicates).

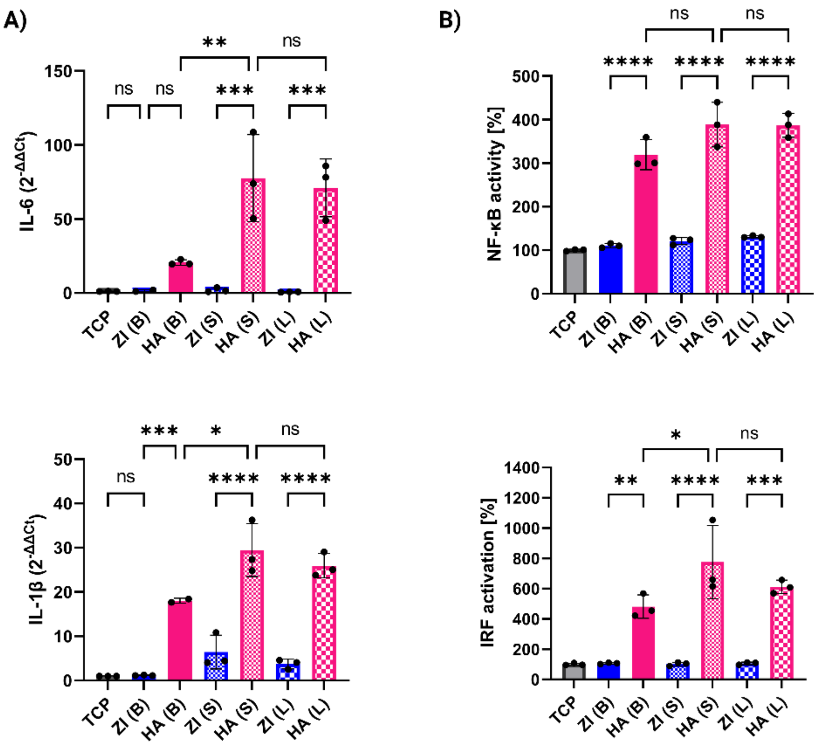

Figure S8. ***In vitro* study with THP-1 derived macrophages. A)** Gene expression of IL-6 and IL-1β for THP-1 derived macrophages seeded on hydrogels for 24 hrs. **B)** SEAP reporter readout for NF-κB and IRF activation for THP-1 derived macrophages seeded on hydrogels for 24 hrs. Data are represented as mean ± standard deviation. Statistical significance was determined using a one-way ANOVA with a Tukey's multiple comparisons test (non-significant (ns) p> .05, ∗p < .05, ∗∗p < .01, ∗∗∗p < .001, and ∗∗∗∗p < .0001). (n = 3 replicates).

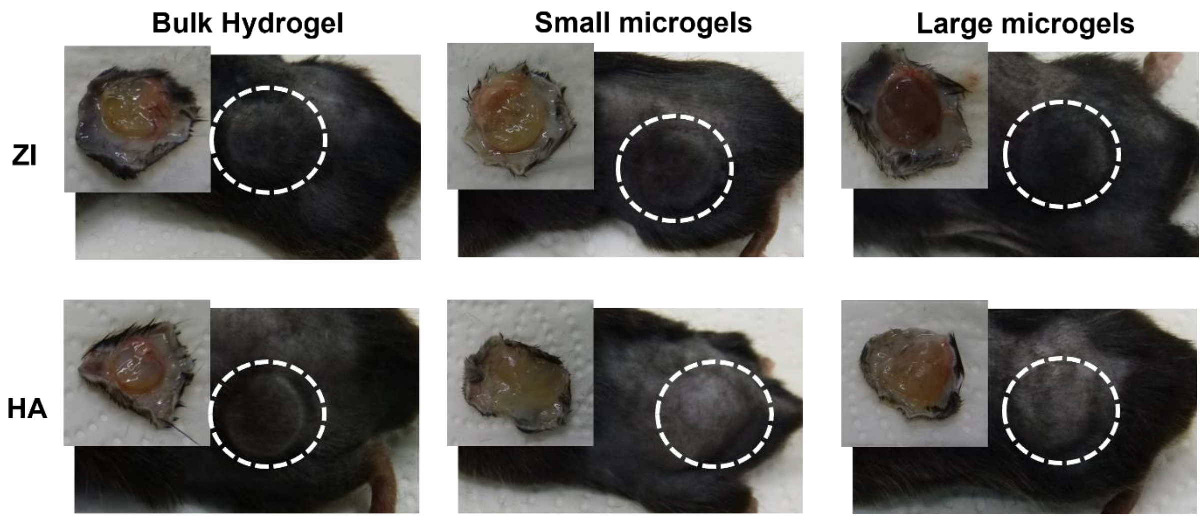

Figure S9. Images from implants under the skin in the back of mice after 10 weeks of implantation, and the harvested samples together with the adjacent skin.

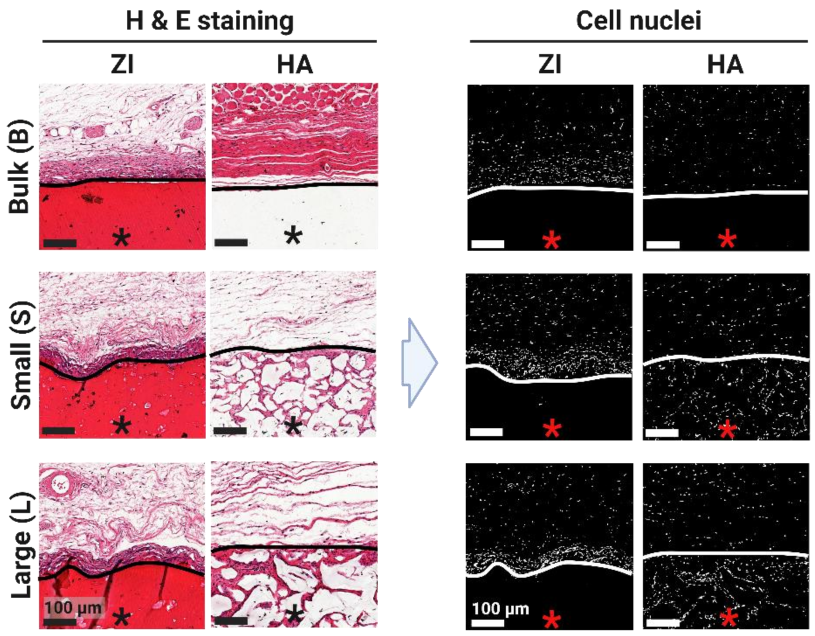

Figure S10. Host cell population around and inside the implants 10 weeks after subcutaneous implantation. A) Representative hematoxylin and eosin (H&E) staining images; black line indicates hydrogel interface with animal tissue, asterisks (*) indicate the location of the implanted hydrogel. (scale bars: 100 μm) **B)** Deconvoluted images from H&E staining showing cell nuclei distribution at the implant interface with animal tissue as well as inside the implants (scale bars: 100 μm).

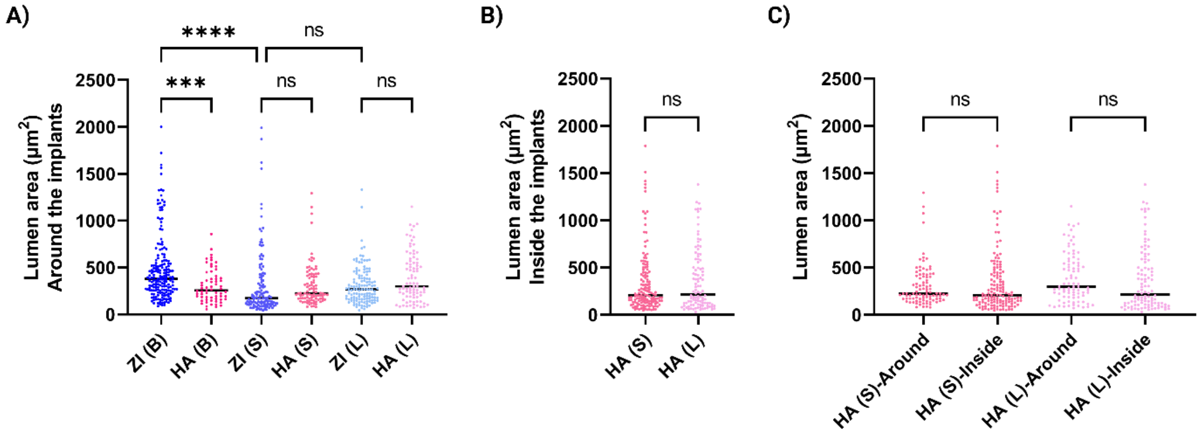

Figure S11. *In vivo* lumen area characterization. Individual lumen area **A)** around the implants and **B)** Inside the HA granular hydrogels. **C)** Comparison of lumen area around and inside HA granular hydrogels. Data are represented as mean ± standard deviation. Statistical significance was determined using a one-way ANOVA with a Tukey's multiple comparisons test (A and C) and unpaired t-test (B). (non-significant (ns) $p > .05$, , $***p < .001$ and $****p < .0001$). ($n = 4$ replicates).

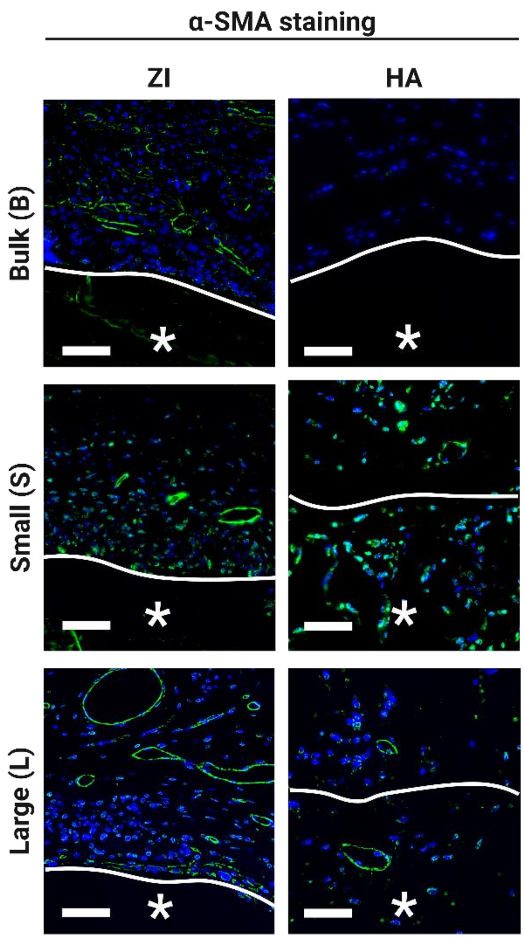

Figure S12. Representative α-SMA immunostaining images of hydrogels (Scale bars: 50 µm; white line indicates hydrogel interface with animal tissue and asterisks (*) indicate the location of the implanted hydrogel).
